# “I know what to say now” Students’ evaluation and utilisation of Accidental Counsellor Training

**DOI:** 10.1371/journal.pone.0333879

**Published:** 2025-10-06

**Authors:** Joanna E. Harnett, Claire E. Ashton-James, James Kite, Tonia Crawford, Sanetta H. J. du Toit, Leigh A. Wilson, Rosa Howard

**Affiliations:** 1 Sydney Pharmacy School, Faculty of Medicine and Health, The University of Sydney, Sydney, New South Wales, Australia; 2 Sydney Medical School, Kolling Institute, Faculty of Medicine and Health, The University of Sydney, Sydney, New South Wales, Australia; 3 Sydney School of Public Health, Faculty of Medicine and Health, The University of Sydney, Sydney, New South Wales, Australia; 4 Sydney Nursing School, Faculty of Medicine and Health, The University of Sydney, Sydney, New South Wales, Australia; 5 Occupational Therapy, School of Medicine and Health Sciences, Edith Cowan University, Joondalup, Western Australia, Australia; 6 Centre for Disability Research and Policy, Faculty of Medicine and Health, The University of Sydney, Sydney, New South Wales, Australia; 7 Sydney Medical School, Faculty of Medicine and Health, The University of Sydney, Sydney, New South Wales, Australia; 8 Graduate School of Medicine, Faculty of Science Medicine and Health, University of Wollongong, Shoalhaven Campus, Mundamia, New South Wales, Australia; University of Ghana College of Humanities, GHANA

## Abstract

**Introduction:**

In 2020 and 2021, 280 health profession students who were engaged in student leadership roles and peer mentoring were invited to undertake extracurricular training in accidental counselling to build skills in recognising and responding to a person in distress. The aim of this study was to evaluate the acceptability of the online training course, and to explore students’ confidence to use Accidental Counsellor tools to support peers in distress after completion of the training course.

**Methods:**

A retrospective cohort study including a post- intervention only design was used. A survey was administered approximately 12 months after 225 students completed an Accidental Counsellor training program. The survey instrument assessed reluctance or confidence to recognise and respond to someone having a mental health crisis, and acceptability of the training. Reliability coefficients were computed for scales, and descriptive analyses including frequencies and percentages were conducted using IBM SPSS Statistics (Version 27). Open-text responses underwent descriptive content analysis.

**Results:**

Sixty-one students completed the online survey. Of these, 90% felt confident and 85% felt low reluctance to respond to peer mental health crises. Nearly all (85%) participants recognised at least one person in distress in the 12 months following the training. T-test confirmed no gender differences in mean confidence score (Men mean = 30.0, Std. dev. = 2.90; Women mean = 29.5, std. dev. = 4.80; t(63)=0.48, p = 0.633), however, there was a statistically significant difference between age groups as determined by one-way ANOVA (F(2,62)=7.43, p = 0.001). Listening non-judgmentally and referring the person to resources was the most common response to peer distress. The online format was described as offering accessibility and interaction benefits but lacked realism and engagement for some.

**Conclusion:**

With ever increasing rates of depression and other mental health conditions being reported among university students, there is increasing pressure to develop strategies to support mental health and wellbeing. This project is unique in evaluating an online delivery of Accidental Counsellor training. Since completing the training, most participants recognized at least one person in distress and reported confidence and skill to respond.

## 1. Introduction

Mental health disorders (MHD) are prevalent across the world with one in five Australians, reporting a common mental disorder in the past year. MHD are one of the largest contributors to the global burden of disease and represent 32% of years lived with disability from all causes [1,2]. Despite the burden of MHD [3], there has been little improvement in mental health outcomes in many countries, including Australia [4]. The Australian Productivity Commission’s Draft Report [4] acknowledges the current mental health care system does not adequately meet the needs of those living with a mental health disorder. People between the ages of 15 and 25 years of age account for nearly 30% of the staggering 800,000 people across our world who die by suicide each year [5]. It has been reported that only one third of people in this age group actively seek help for their MHD [6].

The largest age group represented in public and private not-for-profit Australian universities are 18–25 year olds [7]. The impact of COVID-19 on the mental health of this age group is yet to be fully realised. A study conducted at the University of California reported an increase in students’ MHD throughout the pandemic with the prevalence of major depressive disorder in 2020 among students being double of that reported in 2019. Furthermore, the prevalence of generalized anxiety disorder was 1.5 times higher in 2020 than in 2019 [8]. National and international organisations recognise that proactive approaches at multiple levels are required to address the levels of stress, and MHD that students are experiencing [9].

Due to the high prevalence of under treated mental illness, there have been efforts by a range of large organisations to train non-health professionals (lay people) to provide basic counselling and first aid level of care to people experiencing mental illnesses and mental health crises. Mental Health First Aid (MHFA) has become a popular training for non-mental health professionals and interested community members. Research involving school students suggests MHFA is effective in increasing recognition of and intentions to assist a suicidal peer [10]. Accidental counselling is one such training provided by national crisis support and suicide prevention organisations like Lifeline Australia. Accidental Counsellor training is aimed at those who would like to be able to safely and effectively support friends, family, colleagues, and strangers who are in distress or experiencing a crisis [11]. The skills learnt in the Accidental Counsellor course can be applied in many contexts. However, formal evaluations of Accidental Counsellor training beyond quality assurance feedback at the point of delivery are lacking. More research is needed to understand how often those who have received training use their skills in the following months and years, and how confident they feel when communicating with a person who is experiencing emotional distress. We hypothesised that training in ‘accidental counselling’ that contains self-efficacy skills and evidence-based content delivered, using self-reflection exercises and active learning, would result in students reporting confidence in their ability to recognise and respond to peers and members of their communities who are in distress.

### 1.1. Aims and objectives

The aim of this study was to evaluate indicators of students’ confidence to support peers in distress following a training course in accidental counselling.

The specific objectives were to 1) report on the self-reported confidence of participants to administer accidental counselling 2) report on whether participants had used their accidental counselling skills and 3) to report any open-ended feedback about the training.

## 2. Materials and methods

### 2.1. Design

A retrospective single group post-intervention only design was used [12]. A survey was administered approximately 12 months after students completed an Accidental Counsellor training program. The survey included rating scales and open-response items assessing participants’ self-reported reluctance, confidence, and use of Accidental Counsellor training behaviours [12].

### 2.2. Procedure

From 2020 to 2021, an email was sent to a total of 280 students (>18 years of age) within the Faculty of Medicine and Health at The University of Sydney, Australia who were involved in peer mentoring and student facing leadership roles. The email invited them to express their interest in undertaking extracurricular training in accidental counselling. Across the two years, 225 students participated in the training program which was designed, organised and led by an external mental healthcare organisation, Lifeline Australia [11].

The Accidental Counsellor training program involved a total of five hours of students’ extracurricular time. The students were required to complete a 1.5-hour self-paced learning module prior to attending a 3.5-hour online workshop facilitated by a Lifeline psychologist. A maximum of 20 students could enrol in a training session. Breakout rooms were used over the three hours to enable groups of four students to participate in guided exercises and role plays that aligned with the aims of Accidental Counsellor training. These aims included: equipping individuals to recognise a crisis through understanding what and how different life events and can trigger distress or a crisis; to respond with confidence by learning to listen and supporting a person by using basic counselling skills, including asking about suicide; to refer a person in distress to a suitable service; and reflecting on self-care through being aware of one’s own stress levels and what works to alleviate them.

On the 1^st^ October 2021, approximately 12 months after completing the extracurricular program, the 225 students were contacted again via their student email and invited to participate in the study. The email explained they were being contacted because they had previously completed this training. The main purposes of the study were to understand how they used Accidental Counsellor training in their day to day lives and collect feedback about the training that would inform training further students. The email contained a link to an anonymous online survey hosted by the platform Qualtrics [13]. Following the initial email invitation, two reminder emails were sent out two weeks apart (15^th^ October and the 31^st^ of October). A participant information statement (PIS) was attached to the email invitations, and it was embedded on the landing page of the survey. The PIS outlined important information about the research including any benefits or risks and contact details of the lead researchers and mental health support services. Written consent was then obtained via ‘tick box’ at the end of the participant information statement, where participants were asked to acknowledge they had read, understood, and agreed to their responses being used for the purposes outlined in the PIS.

### 2.3. Ethical approval and consent

The study protocol was approved by The University of Sydney Human Research Ethics Committee (approval number 2021/604).

### 2.4. Survey measures

The survey instrument was adapted from a previous study evaluating mental health first aid training in a cohort of pharmacy professionals [14]. This survey was chosen because it evaluated a similar program to the Accidental Counsellor program, i.e., mental health first aid and in a cohort that was professionally similar to ours. The adaption included replacing the term mental health first aid (MHFA) with Accidental Counsellor training, and several items were revised for the context of our student cohort. These adaptions are outlined in S1 File.

#### 2.4.1. Reluctance to engaging in a mental health crisis.

The first section of the survey included six items evaluating reluctance to respond to someone who is having a mental health crisis. Participants indicated their level of agreement or disagreement on a five-point Likert scale (1 = strongly disagree to 5 = strongly agree) to the following statements: (1) ‘*I am equipped to respond to someone in a mental health crisis’*, (2) *‘I am equipped to respond to someone who is having suicidal thoughts*’, (3) ‘*I would not force someone in a mental health crisis* to *seek help’*, (4) ‘*I cannot relate to why people contemplate suicide*’, (5) ‘*I am too busy to spend time talking with someone about their mental health’, and (6)* ‘*I do not know most students well enough to know when they are in a mental health crisis’*. We dichotomised response categories as agree and not agree (statements 1, 2, and 3) or disagree and not disagree (statements 4, 5, and 6).

#### 2.4.2. Confidence in ability to recognise, respond and refer a person in crisis.

Confidence in Accidental Counselling skills were also assessed using seven items from the previously published mental health first aid questionnaire [14]. Participants were asked to rate their level of agreement or disagreement on a five-point Likert scale (1 = strongly disagree to 5 = strongly agree) to the following statements: I am confident to… *‘recognise the signs that someone may need help’, ‘ask someone if they are thinking about suicide’, ‘listen non-judgmentally to someone experiencing a mental health crisis’, ‘offer basic level information to someone experiencing a mental health crisis’, ‘offer reassurance to someone experiencing a mental health crisis’*, *‘encourage someone experiencing a mental health crisis to seek professional help’,* and *‘encourage self-help strategies for someone experiencing a mental health crisis’*.

#### 2.4.3. Use of Accidental Counsellor skills since training.

Participants were asked to report their best estimate of the number of times that they had experienced nine situations following the Accidental Counsellor training using a frequency scale of zero, once, two times, three times, four times, and greater than five times. The nine items asked how often they had ‘*recognised someone is having a mental health crisis’*, ‘*recognised someone’s behaviour might indicate they are having suicidal thoughts’*, ‘*asked someone about their distressed mood’*, ‘*asked someone if they were considering suicide’*, ‘*listened non-judgementally to someone experiencing a mental health crisis’*, ‘*referred someone to appropriate resources’*, ‘*referred someone to appropriate resources because you were concerned they were considering suicide’*, ‘*engaged with a mental health crisis resource on behalf of someone’*, and ‘*engaged with emergency medical or police services because of someone experiencing a mental health crisis’*. We collapsed the response categories to never and one or more times for all items.

#### 2.4.4. Demographic questionnaire.

Participant were asked to respond to five demographic items including: age bracket (below 25, 25–34, 35+); gender identity (male, female, ‘other – please specify’ or ‘prefer not to answer’); type of course/degree they were studying (dentistry, health science, medicine, nursing, pharmacy, public health, higher degree research); what stage of their course they were in when they completed the Accidental Counsellor Training’ (year 1, 2, 3 or 4); and an item about their living arrangements, i.e., ‘if they lived alone or with family or with friends/shared house.’

#### 2.4.5. Program feedback questions.

The last section of the survey contained three open-response questions probing students for (1) feedback on the online program format, (2) examples of situations in which students had used the skills in practice, and (3) suggestions for further ways in which the university could support student mental health (see S1 File).

### 2.5. Analysis

Descriptive statistics including frequencies and percentages were calculated for all multiple choice and scaled items using IBM SPSS Statistics (Version 27). To aid in analysis, we tested whether the reluctance and confidence items could be combined to produce subscales using reliability testing. The reluctance items did not appear to measure the same latent concept (Cronbach’s α = 0.34) so we analysed these as individual items only. However, we combined the confidence items to generate a confidence score (Cronbach’s α = 0.89). Raw scores of the constitutive questions were summed to produce the score for each respondent. Only participants who had non-missing values for all statements were included. The score was coded such that a higher score indicated higher confidence (range 7–35).

We conducted chi square tests for association to check for differences by age and gender in agreement with reluctance statements and use of Accidental Counsellor skills. We also used a t-test and one-way ANOVA with Tukey post-hoc test to check for differences in confidence score by gender and age respectively.

Open-text responses to the three feedback questions were brief, lacking the richness required for a qualitative analysis, and hence were described [15] narratively, with the aim of reporting all unique perspectives. Exemplar quotes were reported verbatim to characterise each perspective.

## 3. Results

### 3.1. Characteristics of participants

A total of sixty-five students participated in the survey. Of these, 66% (n = 39) identified as women, 76% (n = 45) were less than 35 years of age, and 89% (n = 55) were in the first three years of their courses. Participants were enrolled across a range of medicine and health degrees including pharmacy (18%, n = 11), public health (6.5%, n = 4), medicine (14.7%, n = 9), nursing (11.4%, n = 7), dentistry (3.2%, n = 2), health sciences (21.3%, n = 13), and higher degree research (19.6%, n = 12). Seven participants (11.6%) lived alone. Not all participants responded to every survey question.

### 3.2. Reluctance to respond to a person who is having a mental health crisis

As shown in Table 1, over 85% agreed or strongly agreed that they were equipped to respond to someone in a mental health crisis or to someone who is having suicidal thoughts. Fifty-eight per cent of participants agreed or strongly agreed that they *‘would not force someone in a mental health crisis to seek help’*. Over 90% disagreed with the statements ‘*I cannot relate to why people contemplate suicide*’ and ‘*I am too busy to spend time talking with someone about their mental health*’, while 35% per cent of participants disagreed with the statement *‘I do not know most students well enough to know when they are in a mental health crisis’*. There were no differences by age or gender for any of the statements.

**Table 1:** Reluctance to recognise, respond to a person having a mental health crisis.

	% Agree (n)	% Disagree (n)	Chi square (df)	P value
I am equipped to respond to someone in a mental health crisis				
Overall	90 (60)	–	–	–
Gender				
Men	95 (20)	–		
Women	86 (38)	–	1.17 (1)	0.280
Age (years)				
Under 25	93 (25)	–		
25-34	89 (24)	–		
35+	82 (9)	–	0.95 (2)	0.622
I am equipped to respond to someone who is having suicidal thoughts				
Overall	87 (58)	–		
Gender				
Men	86 (18)	–		
Women	86 (38)	–	0.01 (1)	0.943
Age (years)				
Under 25	85 (23)	–		
25-34	93 (25)	–		
35+	73 (8)	–	2.62 (2)	0.270
I would not force someone in a mental health crisis to seek help				
Overall	58 (39)	–		
Gender				
Men	67 (14)	–		
Women	52 (23)	–	1.20 (1)	0.273
Age (years)				
Under 25	59 (16)	–		
25-34	59 (16)	–		
35+	45 (5)	–	0.71 (2)	0.701
I cannot relate to why people contemplate suicide				
Overall	–	93 (62)		
Gender				
Men	–	90 (19)		
Women	–	93 (41)	0.15 (1)	0.702
Age (years)				
Under 25	–	93 (25)		
25-34	–	93 (25)		
35+	–	91 (10)	0.04 (2)	0.982
I am too busy to spend time talking with someone about their mental health				
Overall	–	91 (61)		
Gender				
Men	–	90 (19)		
Women	–	91 (40)	0.00 (1)	0.955
Age (years)				
Under 25	–	89 (24)		
25-34	–	96 (26)		
35+	–	82 (9)	2.15 (2)	0.341
I do not know most students well enough to know when they are in a mental health crisis.				
Overall	–	35 (23)		
Gender				
Men	–	25 (5)		
Women	–	40 (17)	1.27 (1)	0.260
Age (years)				
Under 25	–	22 (6)		
25-34	–	52 (14)		
35+	–	22 (2)	5.96 (2)	0.051

### 3.3. Confidence in ability to identify and respond to a person in crisis

As shown in Table 2, over 90% of respondents agreed or strongly agreed with 5 out of 7 statements regarding their confidence to respond to a person in crisis, including confidence in their ability to recognise the signs that someone may need help, listen non-judgementally to someone experiencing a mental health crisis, offer reassurance to someone experiencing a mental health crisis, offer basic level information to someone experiencing a mental health crisis, and encourage someone experiencing a mental health crisis to seek professional help. Eight-three per cent of respondents expressed confidence in their ability to encourage self-help strategies for someone experiencing a mental health crisis. Seventy-seven per cent of respondents reported confidence in their ability to ask someone in a mental health crisis if they are contemplating suicide.

**Table 2:** Confidence in ability to recognise, respond and refer a person who is experiencing a mental health crisis.

Statement I am confident I can:	% Agree (n)
recognize the signs that someone may need help	91 (59)
ask someone if they are thinking about suicide	77 (50)
listen non-judgmentally to someone experiencing a mental health crisis	97 (63)
offer basic level information to someone experiencing a mental health crisis	92 (60)
offer reassurance to someone experiencing a mental health crisis	92 (60)
encourage someone experiencing a mental health crisis to seek professional help	92 (60)
encourage self-help strategies for someone experiencing a mental health crisis	83 (54)

T-test confirmed there was no difference in mean confidence score between men (mean = 30.0, Std. dev. = 2.90) and women (mean = 29.5, std. dev. = 4.80; t(63)=0.48, p = 0.633). There was, however, a statistically significant difference between age groups as determined by one-way ANOVA (F(2,62)=7.43, p = 0.001). Post hoc testing showed that participants aged 35 years or older had significantly lower mean confidence scores (25.7 95%CI 20.7–30.8) compared to both under 25-year-olds (29.8 ± 95%CI 28.7–30.9, p = 0.014) and 25- to 34-year-olds (31.1 95%CI 30.1–32.1, p < 0.001). There was no statistically significant difference between the under 25s- and 25–34-year-olds (p = 0.443).

### 3.4. Use of accidental counselling skills in practice

As shown in Table 3, use of accidental counselling skills was very common. Nearly all participants (85%) recognized someone as having a mental health crisis at least once during the 12-month period following Accidental Counsellor training, including 20% who recognised a mental health crisis four or more times. Just over 50% of respondents recognized someone’s behaviour might indicate they were having suicidal thoughts at least once during the period following Accidental Counsellor training. Over 90% reported asking someone about their distressed mood and 89% listened non-judgmentally to someone experiencing a mental health crisis at least once since the Accidental Counsellor training. Forty-three per cent of respondents asked someone if they were considering suicide at least once. Nearly 80% of respondents reported that they had referred someone to appropriate resources at least once, and 43% referred someone to appropriate resources at least once because they were concerned about suicide. Relatively few respondents reported engaging with a mental health crisis resource on behalf of someone (25% at least once) or engaging with emergency medical or police services because of someone experiencing a mental health crisis (12% at least once). There were no differences in use of any skills by age or gender.

**Table 3:** Frequency of self-reported use of Accidental Counsellor training.

Following the accidental counselling training, how many times have you…	% Never (n)	% 1 or more times (n)	Chi square (df)	P value
**Recognized someone is having a mental health crisis**				
Overall	15 (10)	85 (56)		
Gender				
Men	14 (3)	86 (18)		
Women	14 (6)	86 (38)	0.01 (1)	0.943
Age (years)				
Under 25	7 (2)	93 (25)		
25-34	18 (5)	82 (23)		
35+	27 (3)	73 (8)	2.68 (1)	0.262
**Recognized someone’s behaviour might indicate they are having suicidal thoughts**				
Overall	46 (30)	54 (35)		
Gender				
Men	48 (10)	52 (11)		
Women	45 (20)	55 (24)	0.03 (1)	0.870
Age (years)				
Under 25	44 (12)	56 (15)		
25-34	48 (13)	52 (14)		
35+	45 (5)	55 (6)	0.08 (2)	0.962
**Asked someone about their distressed mood**				
Overall	5 (3)	95 (62)		
Gender				
Men	5 (1)	95 (20)		
Women	5 (2)	95 (42)	0.00 (1)	0.969
Age (years)				
Under 25	4 (1)	96 (26)		
25-34	7 (2)	93 (25)		
35+	0 (0)	100 (11)	1.06 (2)	0.588
**Asked someone if they are considering suicide**				
Overall	57 (37)	43 (28)		
Gender				
Men	62 (13)	38 (8)		
Women	55 (24)	45 (20)	0.31 (1)	0.575
Age (years)				
Under 25	67 (18)	33 (9)		
25-34	48 (13)	52 (14)		
35+	55 (6)	45 (5)	1.92 (2)	0.383
**Listened non-judgmentally to someone experiencing a mental health crisis**				
Overall	11 (7)	89 (58)		
Gender				
Men	14 (3)	86 (18)		
Women	9 (4)	91 (40)	0.40 (1)	0.527
Age (years)				
Under 25	11 (3)	89 (24)		
25-34	15 (4)	85 (23)		
35+	0 (0)	100 (11)	1.79 (2)	0.408
**Referred someone to appropriate resources**				
Overall	22 (14)	78 (51)		
Gender				
Men	10 (2)	90 (19)		
Women	27 (12)	73 (32)	2.65 (1)	0.104
Age (years)				
Under 25	22 (6)	78 (21)		
25-34	19 (5)	81 (22)		
35+	27 (3)	73 (8)	0.37 (2)	0.832
**Referred someone to appropriate resources because you were concerned, they were considering suicide**				
Overall	57 (37)	43 (28)		
Gender				
Men	62 (13)	38 (8)		
Women	55 (24)	45 (20)	0.31 (1)	0.575
Age (years)				
Under 25	56 (15)	44 (12)		
25-34	52 (14)	48 (13)		
35+	73 (8)	27 (3)	1.42 (2)	0.491
**Engaged with a mental health crisis resource on behalf of someone**				
Overall	75 (49)	25 (16)		
Gender				
Men	90 (19)	10 (2)		
Women	68 (30)	32 (14)	3.81 (1)	0.051
Age (years)				
Under 25	74 (20)	26 (7)		
25-34	78 (21)	22 (6)		
35+	73 (8)	27 (3)	0.15 (2)	0.928
**Engaged with emergency medical or police services because of someone experiencing a mental health crisis**				
Overall	88 (58)	12 (8)		
Gender				
Men	86 (18)	14 (3)		
Women	91 (40)	9 (4)	0.40 (1)	0.527
Age (years)				
Under 25	89 (24)	11 (3)		
25-34	93 (25)	7 (2)		
35+	82 (9)	18 (2)	0.95 (2)	0.622

### 3.5. Open-text responses

#### 3.5.1. Student perspectives on the online delivery of Accidental Counsellor training: Advantages and disadvantages.

Thirty-three participants (50%) provided free-text responses to the question *‘please comment about the online training format in relation to your ability to engage in real world experiences.’* Content of the open-text responses to the request for feedback on the online delivery of the program described perceived advantages and disadvantages of online training. Sixteen participants suggested avenues for further supporting students to respond to peer mental health crises.

Participants identified advantages of the online training format. Twenty-two participants (34%) described the online training environment as interactive and supportive:

“I thought it was really great. Everyone was encouraged to participate irrespective of the online format and I felt really engaged. I loved how personable it was during the session and we felt supported the whole time discussing something so heavy.” (P69)

“It is a good method to engage and interact with everyone” (P67)

“It was really helpful and it was interactive which was great. I think it is more about further training more than the format. Online makes it easier and more accessible.”(P43)

Eleven participants reflected that the online format was just as valuable as the alternative, face to face format:

“I believe, personally, the online training format was just as effective as an in-person format would have been in giving me the ability to engage in real world experiences.” (P54)

“The zoom sessions were extremely interactive and well run so I don’t think the online format impaired my training.” (P53)

“I think the online format worked well and was just as effective as if it were face to face.” (P33)

“The online format didn’t hinder my understanding of the content nor the emotional impact it had.” (P19)

Others felt that the online format afforded them an enriched learning environment in several respects. For example, three participants noted that the online format allowed them to take notes, which they anticipated they would refer back to “to renew [their] knowledge and skills at any point”. Participants also felt that the online format helped them to focus on the content of the program.

“Online is very good for me as I can focus on the information rather than get distracted by feeling uncomfortable around others.” (P66)

One participant noted that “It was good that we got to have small breakout rooms to have meaningful discussions even though it was online”. (P68)

In addition to facilitating valuable interactions with others, two participants noted that the online breakout discussion groups facilitated peer-learning:

*“*The online format didn’t hinder my understanding of the content nor the emotional impact it had. The ability to have the one-on-one chat room conversations really helped as some of the students themselves helped with my learning.” (P19)

“I did find it easier and more comfortable talking about it through the online format.” (P22)

Two participants expressed concerns that the online format might not fully prepare them for real-life situations involving individuals in mental health crises:

“Simulations with other participants were not really indicative of real people with mental health crises.” (P58)

“In moments where you are physically with a person, you have to be there for them and cannot check the provided resources to perhaps offer the best reference. When you actually appear with someone experiencing a mental health crisis I do not believe the A.C.T [online Accidental Counsellor training] provided enough practice and I have had to rely on personal experience much more.” (P29)

While several participants found the online training format an effective learning environment (as described above), three reported finding it difficult to learn in the online training environment:

“I find it challenging to retain information in the online format. I feel as though face to face would ‘sink in’ more. Especially with role playing.” (P81)

“It would be easier to immerse into the simulation activities in person.” (P64)

“Training in person is much more memorable and therefore effective.” (P65)

#### 3.5.2. Student reflections on the use of Accidental Counsellor training in practice.

Thirty-three (50%) participants provided free text responses to the question, *‘Have there been any circumstances where you have used your Accidental Counsellor training? If so briefly describe here, without identifying any person/s’*. Participants reported using aspects of their training in a variety of contexts, offering support, intervention, education, and encouragement to friends, classmate or fellow students, work colleagues and family members when they were struggling with mental health and emotional well-being:

“I recently had a friend admit that they had planned to take their life but did not follow through as I had reached out to see how they were doing. I think my Accidental Counsellor training really helped me react in an appropriate way and stay level-headed - we were able to have a really transparent discussion and talk about actions that we could take from there to help them.” (P54)

“At the end of a get together last year, a classmate indicated that they were not doing okay that night. I talked to them, checked whether or not they were contemplating self-harm, listened to them, encouraged them to seek help from necessary groups/professionals, and kept in contact with them via text message while they were traveling home. They made it back okay.” (P74)

“Quite a few times when dealing with fellow students feeling low or hopeless and then helping them to guide their thoughts and directing them to resources, they can utilize and pointing them to the GP or urgent care, as needed.” (P31)

“I have a team member at work who is in a supervisory role and his mental health very poor and unstable. I have been personally involved in more than one conversation about it, two incidences where suicide was part of his thought process during a breakdown. I felt equipped to approach a situation or conversation. The training came at an excellent time for me as I had never had to deal with someone experiencing somethings so serious at work which began just after I attended the sessions.” (P69)

“I have used the listening and conversation strategies consistently since the course when talking to friends or family members who are upset.” (P43)

#### 3.5.3. Suggested avenues for further improving support for students’ mental health.

Thirty-eight (58%) participants provided free text responses to the question, *‘What suggestions would you propose for further supporting university students in mental healthcare? What do you still need help with?’* Participant responses emphasised a desire for greater student access to information about mental health and mental health training resources. In particular, 12 participants (18%) suggested that Accidental Counsellor training should be mandatory for all health professions students.

“Options for more people to undergo the training (like more groups a year) would be beneficial across a greater depth of people. I think a lot of people realised that they could do things different.” (P78)

“I think these sessions should be more widely accessible by healthcare students, it’s critical not just for our patients that we see, but for our peers and for our co-workers.” (P69)

Indeed, several participants suggested that mental health (Accidental Counsellor) training should be offered to all students in the Faculty of Medicine and Health, making this training and Accidental Counsellor interventions a norm within the university community.

“It should be PPD [Peer and Personal Development] mandatory, and credit should be given for those who complete the course.”(P40)

“It’s very possible that mental healthcare could be added to the curriculum for health students.”(P29)

“Regular mental health sessions, weeks [of semester] dedicated to student mental health, activities that encourage conversations [about mental health] in tutorial settings.” (P79)

“I think it would be good to continue having these training sessions for university students, and to better educate students and staff in the resources that are available for them to use.” (P92)

“I think this is a great resource that should be more widely offered to students! The impact would be greater if the uptake of this course was higher.” (P54)

## 4. Discussion

In this study, which employed a retrospective single-group, post-intervention-only design, the principal finding was that extracurricular training in Accidental Counselling has the potential to be an effective approach for upskilling students in responding to individuals experiencing a mental health crisis. Students reported low levels of reluctance and high levels of confidence to respond to someone who is having a mental health crisis, and most reported recognising and responding to a peer who was having a mental health crisis across a variety of communities they engaged, following the training. Several respondents suggested the training should be available to all students who are studying medicine and health related disciplines due to the likelihood of encounters, and more broadly as an important resource for addressing student mental health in universities. Feedback on the advantages of the online format included accessibility, ease of interacting with others, and enhanced learning environment while disadvantages included a perceived lack of realism in simulation sessions, and difficulties remaining engaged with sessions. Overall, however, participants were grateful for the opportunity to join the training program which was afforded by the online format.

Relatively few respondents reported reluctance to respond to and refer someone who may be experiencing a mental health crisis. However, approximately one third of respondents expressed agreement with the statement ‘I do not know most students well enough to know when they are in a mental health crisis’, indicating that students may be more confident engaging with a friend who is seen to be having a mental health crisis than a peer who is unknown to them. A similar finding was reported by Witry and colleagues [14]. The authors reported that while the majority of pharmacy students and clinicians in their study expressed willingness to approach someone who appeared to be having a mental health crisis, 21% of respondents expressed concerns about their ability to recognise a mental health crisis in “most students”. Hence, lack of familiarity appears to be a barrier to recognising and responding to someone in a mental health crisis. Research suggests that lack of familiarity can indeed be a barrier to empathy [16]. However, lack of familiarity is a barrier that can be overcome. Studies have found that making a deliberate effort to take the perspective of someone who is unfamiliar (or perceived to be different) to you (i.e., imagining that you are currently in their situation) reduces stigma and increases helping behaviour [17–19]. Students may be more likely to use Accidental Counsellor training in practice if they are made aware of implicit biases that might lead them to feel that they “don’t know” students from certain groups and how to apply perspective-taking strategies that can help them to overcome these biases.

Respondents reported high levels of confidence in their ability to recognise, respond and refer a person who is experiencing a mental health crisis. On the one hand, respondents may be over-estimating their ability to respond to a mental health crisis in practice, consistent with the Dunning-Krueger effect [20] with the implication that there may be a discrepancy between confidence in one’s ability to respond to a mental health crisis and actual behaviour. On the other hand, reported frequency of implementing Accidental Counsellor skills does appear consistent with reported confidence: The majority of respondents report recognising at least one person experiencing a mental health crisis or recognised that someone’s behaviour might indicate they are having suicidal thoughts in the 12 months following their Accidental Counsellor training, and over 90% of respondents reported asking about a person’s distressed mood and listening non-judgmentally at least once in the period following the training. Moreover, the number of respondents who reported recognising suicidal thoughts was approximately equivalent to the number who reported referring someone to appropriate resources because they were concerned about suicide, indicating that in this cohort, reported confidence in their ability to use the Accidental Counsellor skills is associated with their use of these skills in practice.

The frequency with which students reported recognising a student in crisis should be considered within the context of the COVID-19 pandemic. Given that many university classes were being taught online rather than on campus in the period following the Accidental Counsellor training, opportunities to interact with other students and observe behaviours indicative of mental health concerns will have been limited. Yet, the majority of respondents reported observing mood disturbances or other evidence of a mental health crisis at least over a 12-month period. Several respondents described situations where they had provided support to friends, peers, and co-workers who were considering self-harm or suicide. The value of recognising and responding to another’s’ distress cannot be underestimated. Research indicates that peer support for mental health crises can change recipients’ lives; it can increase their sense of hope and control, ability to effect self-care, sense of community belonging, and satisfaction with life [21]. It may even have a positive impact on recipients’ level of depression and psychosis. It would be valuable for future research examining the outcomes of Accidental Counsellor training to evaluate the benefits of the training from the perspective of the recipient: what was the impact of having their mental health crisis recognised by a peer? What was the consequences of the support provided?

Respondents suggested that Accidental Counsellor training would be beneficial for all students of medicine and health. Others suggested that all university students should have access to a truncated Accidental Counsellor training program to increase awareness of mental health crises, and to normalise responding to crisis in peers. While an interactive, group-based online program is unlikely to be scalable, it is certainly plausible that all students at university could complete an autonomous learning online module to provide them with knowledge about how to recognise a mental health crisis and what steps to take to support someone to access the resources and help they need. Before implementing a faculty-wide Accidental Counsellor program for all students, or even before implementing a truncated online learning module, it may be important to evaluate the potential for adverse events to be experienced by Accidental Counsellors and identify strategies to mitigate any negative effects.

Respondents in the current study did not report any adverse events or negative effects of participating in the program or supporting peers, friends, and/or family members in the aftermath of the program. However, the current study did not explicitly measure adverse events. While there is a substantial body of research into the benefits of peer support programs for those who receive the support, very little is known about the experience of those who provide support. A recent qualitative study investigated the experiences of adolescents who had experience providing support to peers in crisis [22]. The participants in this study had no formal peer support or Accidental Counsellor training and were younger than the respondents in the current study (i.e., University students over 18 years of age). However, it is worth reflecting on the study findings: The authors found that adolescents who felt the sole responsibility for supporting the peer through a traumatic event tended to experience a subsequent mental health crisis themselves, while adolescents who “shared the load” of peer support with their own support network (of family and friends) experienced no such adverse outcomes [22]. While this research was conducted in a very different setting (underprivileged, rural dwelling Australian adolescents versus urban University educated adults), the results raise the possibility that it may not be safe or responsible for universities to mandate Accidental Counsellor training for all students, without consideration of the social supports available to them which may be essential for mitigating vicarious trauma.

### 4.1. Limitations

This study has several limitations that should be addressed in future research. Due to the retrospective single-cohort study design where the survey was administered following and not prior to the training, it is not known whether the Accidental Counsellor training changed students pre-training beliefs or behaviours and reluctance to engage in such encounters. Further to this, our findings may not be representative of university students more broadly as they were selected from a group of high-achieving and motivated group of students who already acted as peer mentors and student leaders and the response rate to our survey was relatively low at 27%. An additional limitation of the study is that the “reluctance” survey items demonstrated low levels of variance, indicating a lack of sensitivity, and behavioural data were based on self-report and as such positive outcomes may be magnified by social desirability bias.

### 4.2. Future research directions

Based on the results of this present research, as well as limitations, we suggest several areas for future research. First, there is a need for pre- and post-survey data, at a minimum, to detect changes in attitudes and behaviour following Accidental Counsellor training. A larger randomised controlled trial would enable researchers to determine that the cause of changes in participants is the training process, as opposed to natural history (life experience, maturation). Second, there is a need to collect observational rather than self-report data to check the fidelity of participants’ use of Accidental Counsellor training skills in practice. Third, we recommend following up with recipients of accidental counselling, where possible and with consent, to develop insight into the impact of the intervention on their mental wellbeing in the short term. Fourth, we recommend investigating whether it is feasible and acceptable to supplement the Accidental Counsellor training with education and training on implicit bias and how to overcome feelings of interpersonal distance using perspective taking. Finally, we recommend evaluating adverse events or negative effects on the wellbeing of accidental counsellors themselves.

## 5. Conclusion

With ever increasing rates of depression and other mental health conditions being reported among university student cohorts, there is increasing pressure on universities to develop scalable strategies to support student mental health and wellbeing. Accidental Counsellor training delivered online has the potential to upskill students to be able to identify and respond to mental health crises in their peers. Twelve months following participation in online Accidental Counsellor training, participants in the current study reported low levels of reluctance and high confidence to recognize and respond to peer mental health crises, and most reported utilizing the skills they learned in the program at least once to support fellow students, friends, family members, or co-workers. Overall, the online format of the training was perceived to be acceptable and appears to be a feasible method of delivering important knowledge and skills to students to enable them to support their peers when in crisis. Future research is needed to measure the effectiveness of the training program and outcomes for accidental counsellors as well as those who are supported.

## Supporting information

S1 File Survey instrument. (CSV)
